# Regulation of Tumor Progression by Programmed Necrosis

**DOI:** 10.1155/2018/3537471

**Published:** 2018-01-31

**Authors:** Su Yeon Lee, Min Kyung Ju, Hyun Min Jeon, Eui Kyong Jeong, Yig Ji Lee, Cho Hee Kim, Hye Gyeong Park, Song Iy Han, Ho Sung Kang

**Affiliations:** ^1^Department of Molecular Biology, College of Natural Sciences, Pusan National University, Pusan 609-735, Republic of Korea; ^2^DNA Identification Center, National Forensic Service, Seoul 158-707, Republic of Korea; ^3^Nanobiotechnology Center, Pusan National University, Pusan 609-735, Republic of Korea; ^4^The Division of Natural Medical Sciences, College of Health Science, Chosun University, Gwangju 501-759, Republic of Korea

## Abstract

Rapidly growing malignant tumors frequently encounter hypoxia and nutrient (e.g., glucose) deprivation, which occurs because of insufficient blood supply. This results in necrotic cell death in the core region of solid tumors. Necrotic cells release their cellular cytoplasmic contents into the extracellular space, such as high mobility group box 1 (HMGB1), which is a nonhistone nuclear protein, but acts as a proinflammatory and tumor-promoting cytokine when released by necrotic cells. These released molecules recruit immune and inflammatory cells, which exert tumor-promoting activity by inducing angiogenesis, proliferation, and invasion. Development of a necrotic core in cancer patients is also associated with poor prognosis. Conventionally, necrosis has been thought of as an unregulated process, unlike programmed cell death processes like apoptosis and autophagy. Recently, necrosis has been recognized as a programmed cell death, encompassing processes such as oncosis, necroptosis, and others. Metabolic stress-induced necrosis and its regulatory mechanisms have been poorly investigated until recently. Snail and Dlx-2, EMT-inducing transcription factors, are responsible for metabolic stress-induced necrosis in tumors. Snail and Dlx-2 contribute to tumor progression by promoting necrosis and inducing EMT and oncogenic metabolism. Oncogenic metabolism has been shown to play a role(s) in initiating necrosis. Here, we discuss the molecular mechanisms underlying metabolic stress-induced programmed necrosis that promote tumor progression and aggressiveness.

## 1. Introduction

Rapidly growing tumors experience hypoxia and nutrient (e.g., glucose) deficiency because of insufficient blood supply. Tumor cells respond to the cytotoxic effects of such metabolic stresses either by activating certain signal transduction pathways and gene regulatory mechanisms to survive or by undergoing cell death, especially in the innermost tumor regions [1–4]. Cell death mostly occurs by necrosis because apoptosis and/or autophagy is limited during carcinogenesis [5–8]. In addition, the development of a necrotic core in cancer patients is correlated with increased tumor size, high-grade disease, and poor prognosis due to the emergence of chemoresistance and metastases. Thus, metabolic stress-induced necrosis plays important roles in clinical implication.

Necrosis has traditionally been considered an accidental and genetically unprogrammed form of cell death. Unlike tumor-suppressive apoptotic or autophagic cell death, necrosis has been implicated in tumor progression and aggressiveness as “a reparative cell death” [5, 9–13]. Necrosis begins with cell swelling, resulting in cell membrane rupture and release of cellular cytoplasmic contents into the extracellular space, such as high mobility group box 1 (HMGB1), which is a nonhistone nuclear protein that regulates gene expression and nucleosome stability and acts as a proinflammatory and tumor-promoting cytokine when released by necrotic cells [14–18]. These released molecules recruit immune cells, which can evoke inflammatory reactions and thereby promote tumor progression by increasing the probability of proto-oncogenic mutation or epigenetic alterations and inducing angiogenesis, cancer cell proliferation, and invasiveness [5, 9–13]. HMGB1 contributes to inflammation, immunity, metastasis, metabolism, apoptosis, and autophagy during tumor development and cancer therapy. HMGB1 plays an important role in regulating epithelial-mesenchymal transition (EMT), which initiates tumor invasion and metastasis. HMGB1-RAGE/TLR2/TLR4-induced EMT appears to be mediated by Snail, NF-*κ*B, and STAT3. The role of HMGB1 is discussed in detail in Section 4.

It has recently been shown that necrosis can also be regulated (such as necroptosis). Oxygen and glucose deprivation- (OGD-) induced necrosis has an important role in tumor progression. However, the regulatory mechanisms underlying metabolic stress-induced necrosis in tumors have been poorly understood. Recently, several regulatory molecules involved in necrosis, including Snail and Dlx-2, have been shown to play important roles in the metabolic reprogramming of cancer cells. Therefore, understanding the precise mechanisms of necrosis-linked tumor progression will be crucial for developing therapeutic strategies.

In this review, we discuss the molecular mechanisms underlying OGD-induced programmed necrosis, which promotes tumor progression and aggressiveness, and how necrosis-induced molecules Snail and Dlx-2 regulate metabolic stress-induced tumor necrosis, focusing on mitochondrial respiration and oncogenic metabolism. We propose that understanding the precise mechanisms of necrosis-linked tumor progression will be crucial for developing therapeutic strategies against cancer.

## 2. Cell Death Modes: Apoptosis, Autophagy, and Necrosis

**Figure 1:**
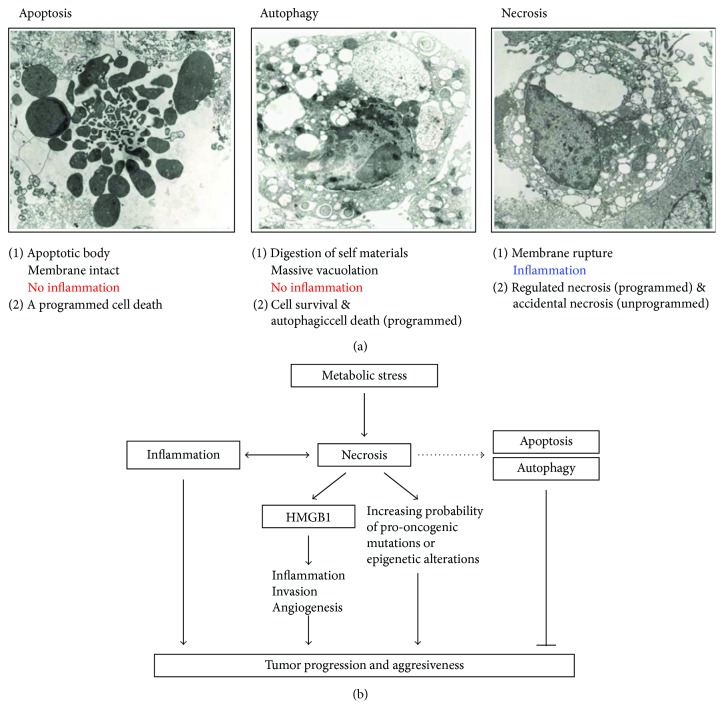
Metabolic stress-induced necrosis is closely associated with tumor progression and aggressiveness. (a) Cell death is generally classified into three categories: apoptosis, autophagy, and necrosis, which are characterized by the distinct biochemical and morphological changes listed. (b) Diagram depicting how necrosis promotes tumor progression and aggressiveness by releasing the proinflammatory and angiogenic cytokine HMGB1, which can contribute to malignancy through increasing the probability of proto-oncogenic mutations or epigenetic alterations.

Multicellular organisms make use of cell death as a tool to eliminate unwanted cells to shape their bodies and regulate tissue homeostasis [19]. Cell death is generally classified into three categories: apoptosis, autophagy, and necrosis [19] (Figure 1(a)).

Apoptosis is a form of programmed cell death. This process includes characteristic events such as cell membrane blebbing, cell shrinkage, nuclear fragmentation, chromatin condensation, and chromosomal DNA fragmentation [20–22]. Apoptotic processes are mediated by two basic signaling pathways: the intrinsic (mitochondrial pathway) and extrinsic pathways (death receptor pathway) [22, 23]. Intracellular stimuli, including DNA damage, growth factor deprivation, and oxidative stress, may activate the intrinsic apoptotic pathway. Extracellular signals, including toxins, hormones, growth factors, nitric oxide, or cytokines, may activate the extrinsic apoptotic pathway through the binding of death ligands [e.g., Fas ligand (FasL), TNF-related apoptosis-inducing ligand (TRAIL), and TNF-*α*] to death receptors of the TNF receptor superfamily [22, 24].

Anoikis is a specific type of apoptosis induced by the loss of contact between a cell and the extracellular matrix. Anoikis mediates cell viability by ECM detachment-induced changes. Cancer cells develop anoikis resistance to survive, and anoikis resistance promotes metastasis [25, 26].

Paraptosis is a type of programmed cell death, which is morphologically and biochemically distinct from apoptosis. It is characterized by the vacuolization of ER components and mitochondria swelling. This process is a type of caspase-independent cell death, and gene transcription and translation are required. Paraptosis lacks several characteristics of apoptotic morphologies, such as membrane blebbing, chromatin condensation, and nuclear fragmentation. Paraptosis is induced by human insulin-like growth factor I receptor (IGFIR) and mediated by the MAPK/ERK and JNK/SAPK pathways [27–30].

Autophagy is an evolutionarily conserved catabolic process, in which cells disassemble unnecessary or dysfunctional cytoplasmic components to renew or remodel them. Autophagy is activated by starvation conditions (including deficiency of nutrients such as amino acids), hypoxic conditions, and high temperatures. Autophagy is essential for cell survival, as it provides energy [31, 32]. Autophagy is critical for regulating cell growth. This process may promote survival under stressful conditions; the breakdown of cellular components promotes cellular survival by lowering cellular energy requirements under conditions of extreme starvation. However, in certain circumstances, autophagy appears to promote cell death and morbidity [24, 33]. Autophagic cell death is another process of programmed cell death. Unlike the energy-providing survival mechanism of autophagy, exacerbated or blocked autophagy appears to promote cell death and morbidity [24, 33]. Autophagy depends on many evolutionarily conserved autophagy-related genes (ATGs), which are dramatically induced by starvation and other stressors. Therefore, ATG proteins (such as Atg5, Atg6, and Atg7) and the formation of autophagosomes contribute to the induction of autophagic cell death and autophagy (cell survival). However, ATG expression levels are quite different during these two processes. In particular, Atg5 and Atg6 protein expression levels are low during autophagy for survival, whereas these protein levels are high during autophagic cell death [31, 32]. Mitophagy is the selective degradation of the mitochondria by autophagy and is important for maintaining cell health. This process prevents the accumulation of dysfunctional mitochondria, which induces cellular degradation following damage or stress. The elimination of damaged mitochondria is mediated by the mitochondrial outer membrane protein autophagy-related gene 32 (ATG32) in yeast. Mitophagy is regulated by a pathway comprised of PTEN-induced putative protein kinase 1 (PINK1) and the E3 ubiquitin ligase Parkin in mammals [34, 35].

In contrast, necrosis is defined as a bioenergetic catastrophe and a disordered cell death. Necrosis is characterized by the swelling of cellular organelles, loss of plasma membrane integrity, random DNA degradation, and uncontrolled release of molecules such as HMGB1 and LDH from the dying cells into the extracellular space, which stimulates immune response or activates wound repair [8]. Necrotic cell death is initiated by a wide range of pathological states, including ischemia, trauma, and infection, and various mediators such as ROS and calcium [8, 36, 37]. In contrast to apoptosis and autophagy, necrosis is generally considered the passive destruction of cell components [38]. However, many recent studies indicate the existence of multiple pathways for regulated necrosis [19], although some of the accidental necrosis may exist as an unprogrammed cell death. Regulated necrosis will be discussed below.

As mentioned above, because necrosis and apoptosis exhibit quite different biochemical characteristics, cells are analyzed using various experimental approaches to distinguish between necrotic cells and apoptotic cells, including Hoechst 33342 (HO)/propidium iodide (PI) double-staining, annexin V (AV)/PI double staining, TUNEL enzymatic apoptosis staining, DNA fragmentation, PARP cleavage, HMGB1 release, and lactate dehydrogenase (LDH) assay.

The loss of plasma membrane integrity observed in cells undergoing necrosis occurs in the absence of nuclear condensation, whereas nuclear fragmentation and chromatin condensation are observed during apoptosis induction. The characteristics of apoptosis and necrosis can be detected by HO/PI double staining, which involves the use of the DNA-binding dye HO, which crosses the plasma membrane of all cells, whether they are damaged or not, causing blue nuclear fluorescence, and PI which only penetrates cells with damaged membranes and leads to red/pink nuclear fluorescence [39]. Thus, intact blue nuclei, intact pink nuclei, condensed/fragmented blue nuclei, and condensed/fragmented pink nuclei are considered to indicate viable, necrotic, early apoptotic, and late apoptotic (secondary necrotic) cells, respectively [40].

Annexin V (AV) is a Ca^2+^-dependent phospholipid-binding protein with high affinity for phosphatidylserine (PS). PS is predominantly observed on the inner cell membrane surface facing the cytosol. Exposure of PS on the external surface of the cell membrane is observed in the early phases of apoptosis during which the cell membrane remains intact. Changes in PS asymmetry, which is analyzed by measuring AV binding to the cell membrane, are detected before morphological changes associated with apoptosis have occurred and before membrane integrity has been lost. As described above, necrotic cell death is detected using PI, a cell-impermeant dye [39]. Double staining cells simultaneously with AV (green fluorescence) and the nonvital dye PI (red fluorescence) allows the discrimination of intact (AV−/PI−), necrotic (AV−/PI+), early apoptotic (AV+/PI−), and late apoptotic (AV+/PI+) cells [40].

Necrosis is characterized by genomic fragments of irregular size and DNA smearing, and apoptosis is characterized by the formation of DNA fragments. DNA fragments can be detected by DNA agarose gel electrophoresis and TUNEL assays. Apoptotic endonucleases not only affect cellular DNA by producing the classical DNA ladder but also generate free 3′-OH groups at the ends of these DNA fragments [41, 42]. These groups are end-labeled by TUNEL apoptosis staining, allowing the detection of apoptotic cells using a molecular biology-based, end labeling, histochemical, or cytochemical technique [40].

In addition, poly (ADP-ribose) polymerase-1 (PARP-1), a 113 kDa nuclear enzyme, functions to repair DNA damage by adding poly (ADP ribose) polymers in response to cellular stress. PARP-1 produces several specific proteolytic cleavage fragments with different molecular weights. These PARP-1 signature fragments are recognized biomarkers for specific patterns of protease activity in unique cell death programs. PARP-1 is cleaved to fragments of 50 kDa and 63 kDa during necrosis and to fragments of 89 kDa and 24 kDa during apoptosis [43].

Because necrosis is characterized by the release of molecules such as HMGB1 and LDH from the dying cells into the extracellular space and this does not occur in apoptosis, necrosis is confirmed by HMGB1 or LDH release assay [8].

Although necrosis was generally considered a passive, accidental, and unprogrammed form of cell death, it was recently discovered to be regulated in processes like regulated necroptosis. Necroptosis acts as an alternative choice of safe cell death in cases where apoptosis cannot be executed due to the presence of caspase inhibitors, such as during viral infection. Necroptosis is specific to vertebrates, and its dysregulation contributes to tissue damage and inflammation [19]. The signaling pathway responsible for carrying out necroptosis is generally understood. Necroptosis is dependent on receptor-interacting protein kinase 1 (RIPK1) and/or RIPK3 activity and its substrate mixed lineage kinase-like (MLKL) [19, 22, 44–47].

Several other examples of programmed necrosis have emerged, such as parthanatos, ferroptosis, oxytosis, NETosis/ETosis, pyronecrosis, and pyroptosis [19]. Parthanatos is caused by the hyperactivation of PARP1 in response to extreme genomic stress such as DNA breaks induced by ultraviolet light, ROS, or alkylating agents, the Ca^2+^ signaling pathway, or posttranslational modifications [19]. The massive PARylation of target proteins results in cellular depletion of NAD^+^ and ATP, leading to a bioenergetic crisis, which causes cell death by regulated necrosis [19, 46, 48–51]. Parthanatos is involved in several diseases, including Parkinson's disease, stroke, heart attack, and diabetes.

Ferroptosis and/or oxytosis, intracellular iron-dependent and oxidative stress-induced programmed necrosis, respectively, are typically observed in the contexts of cancer and neurodegeneration [19]. These pathways are induced by the inhibition of cystine/glutamate antiporter, which exchanges extracellular cystine for intracellular glutamate [19]. Ferroptosis is driven by the loss of activity of the lipid repair enzyme glutathione peroxidase 4 (GPX4) and consequent increase in levels of lipid-based reactive oxygen species (ROS), including lipid hydroperoxides [52, 53]. Ferroptosis is characterized by morphological alterations in the mitochondria, including reduced mitochondrial size, condensed mitochondrial membrane densities, reduced or vanished mitochondria crista, and ruptured outer membrane [19].

NETosis/ETosis is caused by microbial and viral infection and releases of neutrophil extracellular trap (NET)/extracellular trap (ET) in neutrophils, eosinophils, mast cells, and macrophages [46]. Pyronecrosis and pyroptosis are both highly inflammatory forms of cell death and occur most frequently upon infection with intracellular pathogens. Canonical and noncanonical inflammasomes induce pyroptosis through caspase-1 and caspase-11, respectively. Pyroptosis is dependent on caspase-1 and caspase-11, but pyronecrosis occurs independently of these proteins [46].

### 2.1. The Interplay between Apoptosis, Autophagy, and Necrosis

Apoptosis, autophagy, and necrosis are often initiated by the same stimuli and are regulated by similar pathways, including initiator and effector molecules. These three main types of cell death depend on the cellular context. In addition, apoptosis, autophagy, and necrosis often coexisted and co-operate in a balanced interplay and thereby facilitate cellular destruction in a complementary fashion [24, 54]. Various factors, including the energy/ATP levels, the extent of damage or stress, and the presence of inhibitors of specific pathways (e.g., caspase inhibitors), determine if a cell will undergo apoptosis, autophagy, or necrosis [24, 54]. The interplay between apoptosis and necrosis is determined by intracellular ATP levels. Apoptosis is triggered by high ATP levels, whereas necrosis is preferred by low ATP levels. When intracellular ATP levels are depleted, energy-requiring apoptotic cell death is converted into necrosis. However, complete ATP depletion causes an alternative cellular demise, because some level of ATP is required to complete necrosis [48, 55, 56].

TNF-*α* is the best-characterized necrosis-inducing ligand and is associated with mitochondrial ATP production and ROS generation. It induces PARP1 activation, leading to ATP depletion and subsequent necrosis [48, 55]. TNF-*α* induces necrosis or apoptosis depending on the cell type; it induces necrotic cell death in L-M cells but induces apoptosis in F17 cells [57]. In addition, TNF-*α*, which is present during an antiviral immune response, has been shown to induce necrosis rather than apoptotic death. Furthermore, TNF-*α* also induces autophagy through antigen stimulation and starvation to block necroptosis in several cell lines, such as L929 cells, lymphocytes, and cancer cells [58, 59].

A number of death receptors, including FAS [60], TNFR1, TNFR2, TRAILR1 and TRAILR2 [61–63], typically induce apoptosis, whereas necroptosis occurs when apoptosis is blocked by caspase inhibitors or levels of ATP are low. In addition, ATP depletion induces autophagy to maintain energy levels, whereas necroptosis occurs when autophagy fails. In response to metabolic stress such as growth factor deprivation, limitation of nutrients, and energy metabolism, both apoptosis and autophagy are activated [24, 54].

## 3. Necrosis in Tumors

The cells in the inner regions of solid tumors display hypoxia and/or higher rates of aerobic glycolysis, which occurs because of insufficient blood supply; thus, these changes may be exacerbated by oxygen and glucose deprivation (OGD) and induce necrotic death [1, 3, 4, 64]. Ischemic conditions within the core of many solid tumors induce necrotic cell death. Necrosis is typically observed once a growing solid tumor is >4 mm in diameter. The necrotic core regions are very difficult to treat by traditional tumor therapies such as radiation or chemotherapy [65].

Because most tumor cells are genetically limited in apoptotic pathways and prone to necrotic cell death, OGD-induced necrosis is commonly found in the inner region of tumors. In addition, OGD-induced necrosis or/and apoptosis occurs in brain tissue as well as tumors. In ischemic brain tissue, OGD induces necrosis and/or apoptosis. In cerebral ischemic injury, apoptosis occurs at the periphery, and necrosis is found in core regions. Thus, the ratio of OGD-induced necrosis/apoptosis is significantly different between ischemic brain tissue and tumors.

Three-dimensional (3D) multicellular tumor spheroids (MTS) are an *in vitro* model of solid tumors for necrosis studies because they mimic *in vivo* tumors more closely than *in vitro* two-dimensional (2D) culture of cancer cell lines [66, 67]. OGD-induced necrosis occurs in the innermost regions of MTS. MTS exhibit a proliferation gradient, with proliferating cells at the periphery, cell cycle-arrested cells in inner regions, and necrotizing cells in core regions [66, 68–71].

For drug screening, the MTS model is regarded as more stringent and representative than other *in vitro* models because MTS exhibit similarities to *in vivo* conditions such as cell-cell interaction; hypoxia; drug penetration, response, and resistance; and production/deposition of extracellular matrix. In addition, MTS are better controlled than *in vivo* experiments with intermediate complexity, because MTS are identical in structure, morphology, microenvironment, and cellular physiology due to culture from the same cell type under the same external conditions. Thus, it is now commonly accepted that MTS can be used for drug screening [72, 73].

## 4. Necrosis Promotes Tumor Progression

Necrosis has a tumor-promoting potential as “a reparative cell death” (Figure 1(b)). The development of a necrotic core in cancer patients is correlated with increased tumor size, high-grade tumor progression, and poor prognosis, due to the emergence of chemoresistance and metastases [1–3]. In contrast, apoptosis has been known to be tumor-suppressive. In response to substantial levels of DNA breaks and other chromosomal abnormalities, p53, a tumor suppressor gene, directly transactivates many genes acting in the apoptotic pathways for tumor suppression. Autophagy can have both tumor-suppressive and tumor-promoting functions in cancer, depending on the cellular context. Autophagy was initially considered a tumor-suppressive cell death, because it eliminates oncogenic protein substrates, toxic unfolded proteins, and damaged organelles. In fact, defects in autophagy increase oxidative stress, DNA damage, and genomic instability that facilitate cancer initiation and progression, suggesting that autophagy is a tumor suppression mechanism. Alternatively, it can be tumor-promoting by providing substrates for the increased metabolic and biosynthetic demands and suppressing the activation of innate and adaptive immune responses [31, 74, 75].

There are two major causes of tumor promotion by necrosis. First, necrosis releases danger-associated molecular patterns (DAMPs), particularly HMGB-1, into the extracellular space, thereby inducing inflammation and promoting tumor progression. Additionally, chronic inflammation can progress to cancer through a four-step cancer model: (1) chronic inflammation, (2) mutation of tumor suppressor genes, (3) necrosis, and (4) mutation of proto-oncogene(s) [9, 76].

### 4.1. HMGB1

**Figure 2:**
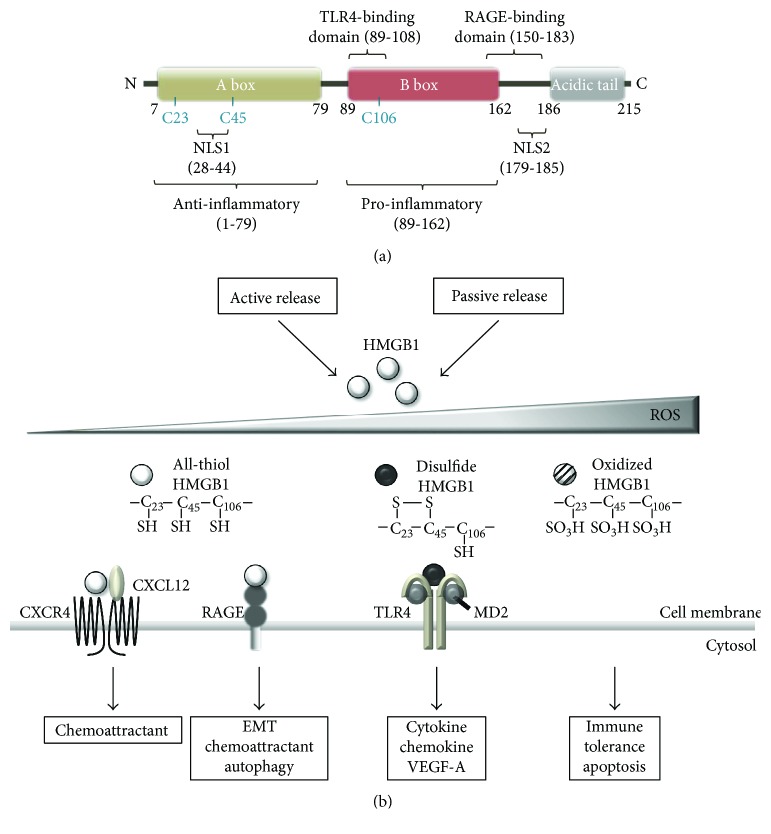
The structure and redox state of HMGB1. (a) Structure of HMGB1. HMGB1 is a-215 amino acid protein, composed of two DNA-binding HMG boxes (a and b) and an acidic C-terminal tail. It contains two nuclear localization signals (NLS) and three conserved redox-sensitive cysteine residues: C23 and C45 in box A, which can form an intramolecular disulfide bond, and C106 in box B. Amino acids 89–108 and 150–183 of HMGB1 are responsible for binding to TLR4 and RAGE, respectively. The A box acts in anti-inflammatory effects, whereas the B box domain plays an important role in pro-inflammatory effects. (b) Redox states of HMGB1 regulate its receptor-binding and extracellular activity. Fully reduced all-thiol HMGB1 (at-HMGB1) has chemoattractant activity. at-HMGB1 forms a heterocomplex with CXCL12 and binds CXCR4, promoting the recruitment of inflammatory cells to damaged tissues. at-HMGB1 binding to RAGE supports its chemoattractant activity via increasing CXCL12 secretion. Disulfide HMGB1 (ds-HMGB1) has sole cytokine activity. ds-HMGB1 induces the release of proinflammatory cytokines via TLR4-mediated signaling. All-oxidized HMGB1 has no cytokine or chemotaxis activity, thereby inducing immune tolerance.

HMGB1, a 215-amino acid protein, is the best-known and characterized DAMP molecule [77–80]. Structurally, HMGB1 is composed of two DNA-binding HMG boxes (A and B) and an acidic C-terminal tail [14–17] (Figure 2). HMGB1 contains two nuclear localization signals (NLS) in box A and between box B and the C-terminal tail [14–17, 81–83]. In the nucleus, it binds DNA without sequence specificity and enables transcription factors, such as p53, p73, the retinoblastoma protein, and estrogen receptor, to access their DNA targets [14–17]. As a DNA chaperone, HMGB1 also participates in DNA replication, recombination, and repair and sustains nucleosome dynamics and chromosomal stability [14–17, 84]. In addition, HMGB1 mediates the transcription regulation of E-selectin, TNF-*α*, BRCA1, and insulin receptor in association with cancer genes [85–89].

#### 4.1.1. HMGB1 as a Damage-Associated Molecular Pattern

When a cell is stressed, damaged, injured, and undergoing necrotic cell death, soluble molecules, such as DAMP molecules, are released on their surface [90]. DAMPs are associated with signaling mediators of sterile inflammatory responses in trauma and injury [91]. They can function as either adjuvants or danger signals by enhancing phagocytosis and antigen presentation and activating the inflammasome [92]. DAMPs act as inflammatory mediators to recruit immune inflammatory cells, which exert a tumor-promoting activity by inducing angiogenesis, cancer cell proliferation, and invasiveness [5, 9–13].

Pattern recognition receptors (PRRs) detect endogenous DAMPs. PRRs initiate the host defense system by recognizing pathogenic components. PRRs include the Toll-like receptor (TLR) family members, the nucleotide binding and oligomerization domain, leucine-rich repeat containing (NLR) family, the PYHIN (ALR) family, the RIG-1-like receptors (RLRs), C-type lectin receptors (CLRs), and the oligoadenylate synthase- (OAS-) like receptors, and the related protein cyclic GMP-AMP synthase (cGAS) [93].

DAMP molecules include alarmins, which are comprised of HMGB1, interleukin- (IL-) 1*α*, IL-16, IL-33, and the Ca^2+^-binding S100 proteins, heat shock proteins (HSPs), ATP, nucleosomes, and mitochondrial components (including mitochondrial DNA) [77, 90, 94–100]. Some alarmin proteins can act with dual functions in the intracellular and extracellular spaces. Alarmins may contribute to beneficial cell housekeeping functions by leading to tissue repair and may cause deleterious uncontrolled inflammation. These dual-function proteins share conserved regulatory mechanisms, including secretory routes, posttranslational modifications, and enzymatic processing [91, 101]. For example, HMGB1 mediates inflammation, cell migration, proliferation, and differentiation as well as chromosomal DNA repair in the extracellular or intracellular space [101]. IL-1*α* enhances local inflammation through activating neighboring fibroblasts or epithelial cells that release chemokines to attract immune cells [101]. IL-16 functions as a chemoattractant and a modulator of T cell activation [91]. When released IL-33 binds to ST2, a member of the IL-1 receptor family (also known as IL-1RL1), IL-33, can activate both innate and adaptive immune cells and then trigger proinflammatory signals or T helper 2 (Th2) cell maturation and response [101]. S100 proteins are also involved in both innate and adaptive immune cells to promote cell migration, proliferation, differentiation, and chemotaxis [101].

HMGB1 as a DAMP is considered one of the hallmarks of immunogenic cell death (ICD). ICD is caused by several cytotoxic agents, such as anthracyclines, oxaliplatin, and radiotherapy [92, 95, 102, 103]. Most of the ICD-inducing agents target the endoplasmic reticulum (ER), leading to ER stress and production of ROS [102, 103]. In addition, 2-deoxyglucose (2DG) is known to cause glycolysis inhibition, synergizing with cytotoxic agents and leading to the induction of ICD [104]. ICD is characterized by the emission of DAMPs, including surface-exposed calreticulin, HMGB1, and ATP. ATP can function as a “find-me” signal, and calreticulin can function as an “eat-me” signal for professional phagocytes. Both HMGB1 and ATP have been shown to trigger the inflammatory reaction [105–108]. HMGB1 secreted by ICD cells has been shown to stimulate cross-presentation by DCs of neoantigens from cancer cells and subsequently activate CD4 T cells that kill tumor cells and establish antitumor immunological memory [105–108]. Thus, HMGB1 could induce an effective antitumor immune response through the activation of DCs [109].

#### 4.1.2. Regulation of HMGB1 Release

In most cells, HMGB1 is usually localized in the nucleus. However, HMGB1 can be released from cells dying by necrosis, apoptosis, and autophagy and by immune cells into the extracellular space [14–17, 81, 110]. In necrotic cells, HMGB1 can be released from the nucleus to the extracellular space to regulate inflammation, angiogenesis, and EMT, which is required for initiating tumor invasion and metastasis. Extracellular HMGB1 decreases the expression of epithelial markers and increases the expression of mesenchymal markers in various cancer cells [17, 111–115]. Since HMGB1 is normally bound loosely to chromatin, it rapidly leaks out of necrotic cells [111, 116]. HMGB1 is also released upon apoptotic cell death and appears to be a tolerogenic response [111–114]. During apoptotic death, HMGB1 irreversibly attaches to chromatin due to histone underacetylation and chromatin condensation. Thus, apoptotic cells release HMGB1-nucleosome complexes, while primary necrotic cells secrete free HMGB1 [111, 114]. If the apoptotic cells are not promptly cleared by phagocytic cells, secondary necrosis may occur, and HMGB1 can leak through the disrupted plasma membrane and induce inflammatory cytokine production [17, 111–115]. Furthermore, HMGB1 can also be released during autophagy [117, 118]. Autophagic stimuli, such as starvation, can promote translocation of HMGB1 from the nucleus to the cytosol in a ROS-dependent manner. Inhibiting autophagy by pretreating with PI3K inhibitors, such as 3-methyladenine (3-MA), prevents H_2_O_2_- or CuZnSOD siRNA-induced HMGB1 release [117]. Extracellular HMGB1 then activates autophagy by dephosphorylating mTOR and upregulating autophagy-associated proteins, Beclin-1 and LC3II [118].

HMGB1 can be actively secreted from activated immune cells in response to exogenous and endogenous stimuli such as bacterial endotoxin, CpG DNA, TNF-*α*, IL-1, and IFN-*γ* and regulates inflammatory responses to infection and injury [119–121]. Immune activators also stimulate the release of HMGB1 via producing secondary messengers, including calcium, ROS, and nitric oxide [14–17].

#### 4.1.3. Functions and Receptors of HMGB1, Depending on Its Redox State

HMGB1 plays a critical, dual role in cancer. Intracellular HMGB1 has been shown to play an important role in maintaining genome stability and autophagy activity during tumor growth; thus, it seems to act as an antitumor protein. This protein suppresses tumorigenesis by preventing chromosome instability-mediated proinflammatory nucleosome release in pancreatic ductal adenocarcinoma (PDAC). However, extracellular HMGB1 presents cytokine, chemokine, and growth factor activity, thus acting as a protumor protein [14, 17, 85, 122]. HMGB1 has been known to play an important role in inflammation, immunity, genome stability, proliferation, metastasis, metabolism, apoptosis, and autophagy [14, 17, 85].

The functions of HMGB1 depend on extensive posttranslational modifications such as acetylation, methylation, phosphorylation, and oxidation. Acetylation and phosphorylation of HMGB1 lead to the translocation of HMGB1 from the cytoplasm and enhance the induction of HMGB1 secretion in macrophages and colon cancer cells [111, 123, 124]. In neutrophils, posttranslational methylation induces the cytoplasmic localization of HMGB1 [125]. HMGB1 is released by a calcium- or ROS-dependent mechanism [120, 126]. Most tumor cells exhibit cytoplasmic localization of HMGB1 and increased HMGB1 expression [127–129].

Once released, HMGB1 stimulates proinflammatory responses through binding to several cell surface receptors, including RAGE, TLR2, TLR4, and TLR9, and activating downstream signaling pathways, such as nuclear factor-kappaB (NF-*κ*B), extracellular signal-regulated kinase 1/2 (ERK1/2), Akt, and interleukin-6 (IL-6)/signal transducer and activator of the transcription 3 (STAT3) pathway [14–17, 81, 82].

**Figure 3:**
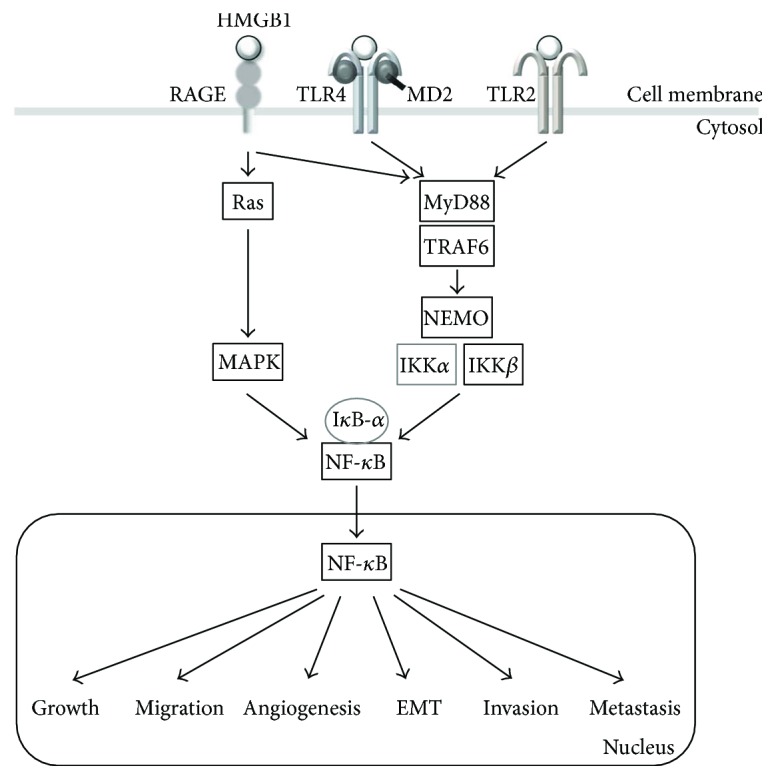
Molecular mechanisms of HMGB1-induced tumor progression and metastasis. HMGB1 is ubiquitous in the tumor microenvironment and functions through activating NF-*κ*B signaling pathways. Extracellular HMGB1 binds to several receptors, including RAGE, TLR2, and TLR4, and activates downstream signaling pathways, such as MAP kinases and myeloid differentiation primary response protein 88- (MyD88-) dependent NF-*κ*B pathways. NF-*κ*B increases expression of its target genes (such as IL-6, IL-8, and Snail) to regulate cancer growth, angiogenesis, EMT, invasion, and metastasis.

HMGB1 contains binding domains for TLR4 and RAGE, encompassing amino acids 89–108 and 150-183, respectively [130, 131] (Figure 2(a)). RAGE, a member of the immunoglobulin gene superfamily of cell surface molecules, was the first reported receptor for HMGB1 [132, 133]. Binding of HMGB1 to RAGE activates Rho family small GTPase (Rac-1, CDC42) and MAPK (ERK, JNK) signaling, leading to the activation of NF-*κ*B [14–17, 81, 82, 134]. RAGE is expressed on immune cells, activated endothelial cells, vascular smooth muscle cells, and many cancer cells. Activation of RAGE contributes to sterile inflammation, cancer, diabetes, and Alzheimer's disease [115]. TLR2 and TLR4 are typical pattern recognition receptors that are expressed on cells of the innate immune system. HMGB1 binding to TLR2 and TLR4 mediates myeloid differentiation primary response protein 88- (MyD88-) dependent activation of the canonical IKK complex and nuclear translocation of NF-*κ*B. This signaling leads to the release of various proinflammatory cytokines, including tumor necrosis factor- (TNF-) *α*, interleukin- (IL-) 1, and IL-6 [114, 135–137] (Figure 3). Therefore, HMGB1 binding to these receptors promotes the recruitment of inflammatory cells, such as neutrophils, monocytes, and macrophages, to damaged sites, and induces the release of proinflammatory cytokines [14–17, 81, 137].

HMGB1 contains three conserved redox-sensitive cysteine residues: C23 and C45 in box A and C106 in box B [14–17, 81, 82] (Figure 2(b)). C23 and C45 can form an intramolecular disulfide bond that stabilizes the folded structures of proteins, particularly those secreted into the extracellular environment [138–140]. In contrast, C106 is unpaired and is necessary for its intracellular translocation [138, 139]. Nuclear HMGB1 mostly exists in a reduced form. Due to the lack of a secretory signal sequence, HMGB1 is secreted via a noncanonical pathway that bypasses the endoplasmic reticulum, an oxidizing environment. Thus, extracellular HMGB1 may exist in both reduced and oxidized forms [140–142] (Figure 2(b)).

The activity of HMGB1 is regulated by its redox states, and the redox state of HMGB1 affects its chemoattractant and cytokine activity [143, 144].

Extracellular fully reduced all-thiol HMGB1 (at-HMGB1) exhibits chemoattractant activity (Figure 2(b)). at-HMGB1 forms a heterocomplex with CXCL12 and then binds to CXCR4 to recruit leukocytes to the injured sites. at-HMGB1 also binds to RAGE to increase CXCL12 secretion [143, 144].

Chemokines fundamentally regulate many cellular, developmental, and physiological processes, including cell activation, differentiation, and trafficking, and are particularly important in leukocyte trafficking. Among chemokines, CXC chemokine 12 (CXCL12), also known as stromal cell-derived factor 1 (SDF-1), is expressed in various cells, including endothelial cells and stromal fibroblasts. When chemokine expression is aberrant, various human diseases, including cancer, can occur [145–149]. Chemokines bind to specific G protein-coupled cell surface receptors (GPCRs) with seven transmembrane-spanning domains. C-X-C chemokine receptor type 4 (CXCR4), a 352-amino acid rhodopsin-like GPCR, specifically binds to the CXC chemokine CXCL12 or SDF-1. Recently, CXCR4 has been shown to play an important role in invasion, angiogenesis, and metastasis, contributing to tumorigenesis and cancer progression. In addition, high CXCR4 expression is observed in a variety of cancers. The interaction between CXCL12 and CXCR4 is involved in many biological processes, including cell survival, proliferation, hematopoiesis, immune response, chemotaxis, migration, and adhesion, through activating various intracellular signaling transduction pathways and downstream effectors [145, 147, 150–153].

Because of the short half-life of at-HMGB1, as time passes, at-HMGB1 undergoes partial oxidation, losing its chemoattractant activity. Disulfide HMGB1 (ds-HMGB1) then binds to TLR4 and activates leukocytes to release proinflammatory cytokines and chemokines [144, 154, 155] (Figure 2(b)). The extracellular TLR4 adaptor molecule, myeloid differentiation factor 2 (MD-2), specifically interacts with ds-HMGB1, allowing for discrimination of different redox states of HMGB1 [154]. C106 is necessary for the interaction between HMGB1 and TLR4. Mutation of C106 inhibits its interaction with TLR4 and prevents macrophage activation and proinflammatory cytokine release [130, 154]. In contrast, all-oxidized HMGB1 has no cytokine and chemotaxis activity, thereby inducing immune tolerance (Figure 2(b)). Oxidized HMGB1 also induces caspase-9/3-dependent apoptosis, resulting in sensitization to cancer therapies [156].

Oxidation by ROS abrogates both chemoattractant and cytokine activities of HMGB1. ROS play an important role in the timely switch from proinflammatory to noninflammatory HMGB1 [144, 154, 155] (Figure 2(b)). ROS can determine whether cell death is immunogenic or tolerogenic by regulating the redox state of HMGB1 [157–159]. In cells undergoing apoptosis, activated caspase-3/7 targets the mitochondria to produce ROS, which leads to the release of oxidized HMGB1, thereby inducing immune tolerance. In contrast, necrotic cells release fully active HMGB1, which activates the immune system. However, at-HMGB1 has a short half-life depending on the extracellular redox environment. Necrotic cells can delay the extracellular oxidation of HMGB1 by releasing cysteine and redox enzymes.

The centers of solid tumors become hypoxic and glucose-depleted, resulting in necrosis and chronic release of HMGB1 [14–17, 81]. This release is prevented by the antioxidant NAC, indicating ROS-dependent release of HMGB1 [160–162]. Consistently, we observed that the overexpression of antioxidant enzymes, manganese SOD and CuZnSOD, suppresses glucose depletion-induced necrosis and HMGB1 release [163]. Because intracellular oxide reductases are often upregulated in the necrotic core, HMGB1 is released with large amounts of these enzymes. Therefore, oxidative stress has been shown to induce HMGB1 translocation and release.

Prolonged extracellular lifespan and activity of HMGB1 can sustain chronic inflammation and contribute to tumor growth, angiogenesis, and metastasis [157–160]. A recent study showed that different redox states of HMGB1 distinctly regulate tumor angiogenesis. at-HMGB1 promotes migration of endothelial cells by interacting with RAGE, whereas ds-HMGB1 binds to TLR4 and activates endothelial cells, stimulating them to secrete proangiogenic factor VEGF-A [164]. In addition, during anticancer therapy, redox states of HMGB1 are also able to regulate the fate of a cancer cell toward cell death or cell survival [139, 156, 165]. at-HMGB1 binds to RAGE, but not with TLR4, to activate Beclin-1-dependent autophagy, thereby promoting resistance to radiotherapy and chemotherapy [156]. Knockdown of RAGE increases sensitivity to chemotherapy via increasing apoptosis and decreasing autophagy [165]. Cytosolic HMGB1 also can induce autophagy through a direct interaction with Beclin-1 via its intramolecular disulfide bridge (C23 and C45) [139]. Thus, inhibiting HMGB1 release or knocking down HMGB1 increases chemotherapy sensitivity in leukemia or pancreatic cancer cells [117, 166]. Therefore, the redox protein HMGB1 plays an important role in tumor progression by regulating immune response, angiogenesis, and the fate of cancer cells in response to anticancer therapy [156, 159, 164].

#### 4.1.4. Regulatory Mechanisms of HMGB1-Induced EMT/Invasion/Metastasis

HMGB1 has been shown to induce epithelial-mesenchymal transition (EMT) in cancer cells. EMT is the process by which polarized epithelial cells are converted to migratory and invasive mesenchymal cells. EMT plays an important role in numerous developmental processes as well as in initiating cancer metastasis [167–170]. Many developmental signaling pathways, such as TGF-*β*, Wnt, Notch, and NF-*κ*B cascades, have been implicated in EMT. The loss of E-cadherin expression is a hallmark of EMT, which can be mediated by several transcription factors, including the Snail, Zeb, and Twist families [170–172]. In addition, the tumor suppressor p53 was found to play a critical role in EMT. p53 downregulates Snail and Zeb1 by inducing the expression of miR-34 and miR-200 family members, respectively [168–170].

Recombinant HMGB1 decreases the expression of epithelial markers, including E-cadherin and ZO-1, and increases the expression of mesenchymal markers, including N-cadherin, vimentin, and *α*-smooth muscle actin in epithelial cells [18, 173–175]. HMGB1-induced EMT appears to be mediated by RAGE [18, 175–178]. Blocking the binding of HMGB1 to RAGE by expressing the HMGB1 150–183 peptide (COOH-terminal motif) inhibits invasive migration of fibrosarcoma cells [131]. Inhibiting the interaction between RAGE and HMGB1 suppresses growth and metastasis of both implanted C6 gliomas and spontaneous tumors *in vivo* [13].

Clinically, RAGE expression has been significantly correlated with metastasis and poor prognosis in many cancer types, including gastric cancer, colorectal cancer, and cervical squamous cell carcinoma [129, 179, 180]. HMGB1/RAGE axis signaling increases the production of key growth factors involved in driving EMT, including TGF-*β*1, platelet-derived growth factor (PDGF), and connective tissue growth factor (CTGF) [177, 178]. HMGB1 enhances TGF-*β* secretion and triggers Smad2/3 phosphorylation, which upregulates Snail, thereby inducing EMT [173, 181]. Knockdown of HMGB1 can suppress TGF-*β*-induced EMT [182]. Furthermore, HMGB1 binding to RAGE is known to activate PI3K/Akt signaling [18, 183]. Activated Akt inactivates GSK3*β*, a known endogenous inhibitor of Snail that results in nuclear translocation of *β*-catenin to induce EMT [18].

HMGB1/RAGE axis signaling also increases phosphorylation of ERK1/2 to induce tumor cell invasion and metastasis [13, 183]. Knockdown of IL-8 suppresses HMGB1-mediated upregulation of ERK1/2 phosphorylation and NF-*κ*B p65 and inhibits EMT [176]. HMGB1 binding to RAGE increases NF-*κ*B expression, phosphorylation, and nuclear translocation to induce EMT and invasion [175, 183, 184]. Activated NF-*κ*B increases the expression of Snail, IL-8, and the IL-8 receptor *β* (IL-8R*β*), which promotes EMT [175, 176]. NF-*κ*B also increases IL-6 expression through activating Lin28-mediated downregulation of let-7 microRNA, which targets IL-6 expression. Subsequent IL-6-mediated STAT3 activation increases miR-21 expression to promote HMGB1-induced invasion and migration [185]. In addition, the RelA/p65 subunit of NF-*κ*B can repress the expression of BRMS1, a metastasis suppressor gene, by promoter-specific methylation in response to TNF [186].

In addition, HMGB1 binding of TLR2 and TLR4 plays an important role in tumor metastasis [160, 187–189]. TLR2 is overexpressed in gastric cancers and colorectal carcinoma [190, 191]. The interaction between HMGB1 and TLR2 promotes the activation of NF-*κ*B, STAT3, and Smad3 and the production of IL-6 and TGF-*β*, leading to breast cancer stem cell self-renewal, tumorigenesis, and metastasis [189]. TLR4 is also overexpressed in melanoma, colon cancer, and breast cancer, and the expression of its downstream adaptor protein MyD88 is elevated in melanoma and colon cancer [192–194]. HMGB1/TLR4-mediated activation of I kappa B kinase (IKK)*β* and IKK*α* signaling contributes to NF-*κ*B activation [195, 196]. The IKK*β*-dependent NF-*κ*B canonical pathway maintains RAGE expression, whereas the IKK*α*-dependent p52/RelB noncanonical pathway sustains CXCL12/SDF1 production to enhance cell migration [195, 196]. Tumor cell-released HMGB1 interacts with TLR4 on platelets, mediating the interaction of tumor cells and platelets to promote metastasis. Thus, TLR4 deficiency impairs this platelet-tumor cell interaction and suppresses experimental lung metastases [187]. A recent study also showed that HMGB1 released from UV-damaged keratinocytes activates TLR4/MYD88-dependent neutrophilic inflammation, which can promote angiotropism and metastasis of melanoma cells [188]. In hypoxic hepatocellular carcinoma cells, HMGB1 binding to TLR4 and RAGE induces caspase-1 activation and proinflammatory cytokine secretion to promote cancer invasion and metastasis [160].

Recent findings indicated that HMGB1 also plays an important role in regulating EMT. HMGB1-RAGE/TLR2/TLR4-induced EMT appears to be mediated by Snail, NF-*κ*B, and STAT3 through the activation of signaling pathways, including TGF-*β*, phosphatidylinositol 3-kinase (PI3K)/Akt, mitogen-activated protein kinase (MAPK), and IKK [13, 18, 131, 175, 176, 183, 184, 197]. We also found that HMGB1 induces EMT via RAGE/TLR2/TLR4-mediated Snail activation. However, the underlying mechanism of HMGB1-induced EMT, invasion, and metastasis remains elusive. Since Snail is implicated in the regulation of glucose metabolism and mitochondrial repression [198, 199], HMGB1 signaling may also be associated with alterations in mitochondrial function and glucose metabolism by Snail-dependent mechanisms. Indeed, HMGB1 regulates cancer metabolism by inducing mitochondrial complex I activity in a RAGE-dependent manner, leading to tumor cell proliferation and migration [200].

In cancer, excessive HMGB1 release is known to contribute to angiogenesis, evasion of programmed cell death (apoptosis), inflammation, tissue invasion, and metastasis [201]. When serum levels of HMGB1 are high, its hyperacetylated and disulfide isoforms have been shown to be sensitive disease biomarkers and contribute to the different disease stages [202]. In patients with various types of cancer, HMGB1 overexpression is correlated with poorer prognosis; HMGB1 overexpression is implicated in poorer overall survival (OS) (HR: 1.99; 95% CI, 1.71–2.31) and progression-free survival (PFS) (HR: 2.26; 95% CI, 1.65–3.10) in a variety of different cancer types. These studies indicate that HMGB1 overexpression is a prognostic factor and potential biomarker for cancer survival [203].

In addition, other nuclear binding proteins like histone H1 subtype (H1.2) are released from necrotic tumor cells and transported into surrounding viable cells and selectively taken up by energy-dependent endocytosis, although the biological relevance of this is not clear [204].

### 4.2. Inflammation and Cancer

Necrotic cells recruit immune and inflammatory cells upon the release of cellular contents, which exerts tumor-promoting activity by inducing angiogenesis, cancer cell proliferation, and invasiveness. The release of HMGB1 by necrotic cells can enhance inflammatory responses, tumor formation, and metastasis through the release of proinflammatory cytokines by activating proinflammatory signaling pathways, including NF-*κ*B and inflammasome pathways [14, 77–80, 201]. HMGB1 is also associated with several inflammatory disorders [115]. Many human cancers are associated with inflammation, and cancer frequently arises in chronic inflammatory states. Chronic inflammation or infectious agents are linked to 25% of all cancers [205]. Among the chronic inflammatory conditions leading to cancer, persistent *Helicobacter pylori* infection induces gastric carcinoma and lymphoma. Hepatitis B and C are linked to diseases including hepatocellular carcinoma, and oral squamous cell carcinoma is induced by lichen planus or gingivitis. Oral squamous cell carcinoma is also associated with chronic periodontitis, and salivary gland carcinoma is induced by sialadenitis [90, 205–207].

Inflammation results in bioactive molecules from immune cells infiltrating the tumor microenvironment, including growth factors to sustain proliferation, prosurvival factors to limit cell death, proangiogenic factors, and extracellular matrix-modifying enzymes to facilitate angiogenesis, invasion, and metastasis, and inductive signals to trigger EMT and other tumor-progressing programs [90, 208–210].

Chronic inflammation can progress to cancer through a four-step cancer model: (1) chronic inflammation, (2) mutation of tumor suppressor genes, (3) necrosis, and (4) mutation of proto-oncogene(s) [9, 76]. Chronic inflammation due to infectious microorganisms or chemicals is associated with lethal forms of cancer [9, 211]. Prolonged chronic subclinical inflammation provides a suitable environment for the progression of clinically apparent cancer [9]. Chronic inflammation can promote cancer development and induce certain cancers and solid tumors. Subsequently, chronic inflammation may initiate and maintain local inflammatory processes that promote tumor progression and dissemination [212–216].

Inflammation is known to play important roles in all stages of cancer development, such as cancer initiation, promotion, malignant conversion, invasion, and metastasis. Inflammatory cells, which exist in the tumor microenvironment or during chronic inflammation, can promote tumor initiation and support tumorigenesis in the extrinsic pathway of inflammation [90, 205–207].

MYC and RAS family oncogenes induce the recruitment of leucocytes and lymphocytes, the expression of chemokines and cytokines, and induce the angiogenic switch, which activates transcription factors in the intrinsic pathway of inflammation-induced cancer. These changes lead to remodeling of the tumor microenvironment [205–207, 217, 218]. An inflammatory microenvironment can enhance mutation rates and increase the proliferation of mutated cells. Activated inflammatory cells induce the production of ROS and reactive nitrogen intermediates (RNI) that may damage DNA and genomic instability in neighboring epithelial cells. The production of cytokines by inflammatory cells also induces the intracellular production of ROS and RNI in premalignant cells to induce epigenetic changes [90].

These factors may cause tumor initiation through DNA damage or genetic instability in inflammation and promote malignant cell transformation by genetic mutations and epigenetic mechanisms in chronic inflammation [219, 220]. Inflammation-related mutagenesis may inactivate or repress the genes of mismatch repair response, and ROS released from inflammatory cells may inactivate mismatch repair enzymes via oxidation [221, 222]. The failure of the mismatch repair system enhances inflammation-induced mutagenesis and inactivates several important tumor suppressors, such as Tgfbr2 and Bax, which harbor microsatellite sequences [90, 221].

The immune defense mechanisms are considered important for inhibiting or promoting cancer and can contribute to cancer progression [90, 205–207]. Myeloid-derived suppressor cells, which are immature myeloid progenitor cells and the major immune suppressor cells, play important roles in tumor progression by activating transcription factors NF-*κ*B and STAT-3 in the tumor inflammatory microenvironment [205]. NF-*κ*B and STAT3 are activated in most cancers and are required for regulating genes involved in tumor proliferation, survival, angiogenesis, invasiveness, motility, and chemokine and cytokine production [223, 224]. The production of cytokines induced by tumor-infiltrating immune cells mediates protumorigenic processes, including survival, proliferation, growth, angiogenesis, and invasion, by activating NF-*κ*B or STAT3 in promalignant cells. In addition, NF-*κ*B and STAT3 induce chemokine production, which recruits immune and inflammatory cell to sustain tumor-associated inflammation [90, 225].

During oxidative stress, low-level production of ROS and RNS leads to cell proliferation, angiogenesis, apoptosis resistance, invasion, and metastasis by the activation of the NF-*κ*B signaling pathway in inflammatory conditions and this induces carcinogenesis [205, 226]. Therefore, an inflammatory microenvironment contributes to protumorigenic microenvironment and these changes are associated with most malignancies [90, 205–207].

Inflammation induces oncogenic mutations that inhibit tumor suppressor genes and activate proto-oncogenes. Knudson proposed “a two-hit model,” which suggests at least two allelic mutations in an individual gene, particularly in tumor suppressor genes, were necessary to cause cancer. These two allelic mutations may cause cancerous growth, and additional mutations (three to seven hits) are necessary for growth-promoting proto-oncogene(s). The mutation of proto-oncogenes contributes to a state of primary genomic instability and a full neoplastic phenotype. Inflammatory processes are associated with the local accumulation of products of cyclooxygenase (COX) activity and nitric oxide and contribute to the epigenetic regulation of genes, cell death, proliferation, and mutagenic hits [9, 76].

Inflammation has been linked to the hypermethylation of tumor suppressor and/or proapoptotic genes. In addition, these changes lead to genetic limitations in apoptotic pathways, which enhances necrotic cell death [9, 227]. Necrotic cell death promotes inflammation through the release of cellular contents, which contributes to cell growth, cancer progression, and tumor-infiltrating leukocyte recruitment [9]. Interestingly, a shift towards necrosis can be triggered by blocking apoptosis in multicellular tumor spheroids. Necrotic cells induce COX-2 expression and then subsequent PGE2 secretion from live tumors, which promotes tumor growth and inhibits activated cytotoxic T cells [228]. This indicates that necrosis is associated with inflammation in tumor progression [9].

## 5. Mechanism of OGD-Induced Necrosis

Oxygen and glucose deprivation- (OGD-) induced necrosis has an important role in tumor progression; however, its regulatory mechanisms have been poorly investigated.

### 5.1. Hypoxia

Hypoxia is a hallmark of solid tumors and is associated with poor prognosis. Hypoxia not only regulates cell survival but also increases the possibility of metastasis, angiogenesis, vasculogenesis, and invasiveness. This process is also involved in altered metabolism and therapeutic resistance [229, 230]. Hypoxia contributes to genomic instability through increasing ROS production and suppressing DNA repair pathways, resulting in therapeutic resistance [229, 230].

Hypoxia raises a crucial signal for cell death. Apoptosis is normally induced under a reduced oxygen supply, since oxygen is required for the function of the electron transport system [231]. However, in tumors, hypoxia-induced apoptosis can be prevented by restoring the oxygen supply by enhancing angiogenesis, hematopoiesis, and levels of intracellular nitric oxide synthase (iNOS), a local vasodilator [232, 233]. Hypoxic cell death can also be avoided by metabolic reprogramming such as enhancing anaerobic ATP production via glycolysis, which is attained by promoting glucose transporter 1 (GLUT1) or pyruvate dehydrogenase kinase (PDK) activity.

In contrast to normal conditions where apoptosis is induced by hypoxia, human glioblastoma cells notably show predominant necrosis but little apoptosis, despite extensive hypoxia. Glioma cells resist cytotoxicity from hypoxia until energy stores are depleted and eventually die via necrosis [234]. Hypoxia-inducible factor-1*α* (HIF-1*α*) is a key transcription factor in the homeostatic response to hypoxia [232, 235]. HIF-1 is a heterodimer composed of an oxygen-sensitive *α* subunit and a constitutively expressed *β* subunit. Under normoxia, HIF-1*α* is rapidly degraded, whereas hypoxia induces stabilization and accumulation of HIF-1*α* [236–239]. Several mechanisms are known to induce HIF-1 activation by increasing the translation of HIF-1*α* mRNA or inhibiting HIF-1*α* degradation; levels of HIF-1*α* mRNA are enhanced by activation of the PI3K/Akt/mammalian target of rapamycin (mTOR) pathway and by the binding of YB-1, an RNA- and DNA-binding protein. HIF-1*α* protein degradation is prevented by ROS and NO. Inactivation of von Hippel-Lindau tumor suppressor protein (pVHL, an E3 ubiquitin ligase targeting HIF-1*α*) and activation of WSB1 (an E3 ligase targeting pVHL) and ubiquitin C terminal hydrolase-L1 (UCHL1, a HIF-1 deubiquitinating enzyme) are also known to induce HIF-1*α* stabilization and activation [236–239].

Under hypoxia, HIF translocates into the nucleus, binds typically to a hypoxia response element (HRE) in the upstream promoter region of its target genes, and affects their transcription to regulate angiogenesis, apoptosis, metabolism, and cell survival [232, 235, 240, 241]. Hypoxia increases ROS production, which sustains the drive into cellular dysfunction and death. Disrupted redox homeostasis predisposes breast cancer stem cells (BCSCs) to ROS-induced cell death [242]. HIFs are important for maintaining BCSCs; HIFs may directly transactivate genes encoding pluripotency factors including FOXO3 and NANOG, under hypoxic conditions, promoting cell survival [242, 243]. Glucose metabolism is also reprogrammed by hypoxia in breast cancer cells to maintain redox homeostasis and cell survival [242, 244, 245].

HIF-1*α* is an important antiapoptotic factor [231, 246, 247]. HIF-1, like tumor necrosis factor- (TNF-) *α*, activates the expression of FoxM1, which promotes the growth of cancer cells in the liver by bypassing apoptotic pathways [248]. HIF-1 overexpression in liver cancer limits the expression of various caspases and of Bax and Bak, leading to a higher intracellular concentration of cytochrome C [232]. Hypoxic apoptosis can be also inhibited by increased expression of survivin and Bcl-family proteins, which are important factors that cause DNA fragmentation [232, 249]. HIF-1*α* and its target genes regulate several biological processes under hypoxia. The transcription factor SP-1, the cytokine interleukin- (IL-) 8, and the growth factor PDGF are target genes of HIF-1*α*.

Stimulating protein-1 (SP-1) is a hypoxia-inducible gene and a sequence-specific DNA-binding protein, and its levels are increased in hypoxic conditions. In hypoxia, SP-1 also induces the expression of cyclooxygenase-2 (COX-2), an inflammation-associated enzyme. In addition, SP-1 mediates the regulation of gene expression by the Sp1–Smad3–HIF-1 complex to activate gene expression in cooperation with HIF-1 [250].

Expression of interleukin-8 (CXCL-8, IL-8), a key factor of endothelial cell survival and angiogenesis, is driven by hypoxia to directly control endothelial cells [251–254]. IL-8 also regulates pathological angiogenesis, tumor growth, and metastasis [232, 253]. HIF-1*α* knockdown directly represses tumor growth, whereas IL-8 knockdown does so indirectly [255–257]. Combined knockdown of HIF-1*α* and IL-8 is more effective in hindering angiogenesis by HCC cell-conditioned media on tube formation and invasion by endothelial cells *in vitro* and increases survival rates of mice as well [232, 256]. IL-8 plays a role in tumor apoptosis by controlling apoptosis in blood vessels and also potentiates the tumor maintenance caused by HIF. Inhibiting HIF-1*α* and IL-8 upregulates the expression of apoptotic factors while simultaneously downregulating antiapoptotic factors [232, 256].

Platelet-derived growth factor (PDGF), a potent mitogen, is important in embryonic development, and its overexpression contributes to different types of malignancies. PDGF also is closely linked to the VEGF expression and is involved in promoting tumor growth, invasion, and angiogenesis [258, 259].

### 5.2. GD

Glucose deprivation (GD) can induce either apoptosis or necrosis, depending on the cell type, due to their different cellular contexts; GD induces necrosis in A549, HepG2, and MDAMB-231 cells, while it induces apoptosis in HeLa and HCT116 cells [40, 71, 260].

#### 5.2.1. Role of ROS and Mitochondria in GD-Induced Necrosis

The cellular functions of ROS are quite diverse in cancer, depending on the identity of ROS and the location of its generation, as well as the local concentration [261]. Weak doses of hydrogen peroxide and superoxide promote cell proliferation in a wide variety of cancer cell types, in contrast to high doses [261]. In addition, ROS can induce either apoptosis or necrosis in human cancer cells, according to the level of insult; low levels of ROS induce apoptosis, whereas higher levels induce necrosis [262]. These suggest that ROS play important roles in cancer cell proliferation and cell death.

Excess ROS production, mitochondrial dysfunction, and ATP depletion are involved in necrosis [8, 263]. Mitochondrial ROS is closely linked to GD-induced cytotoxicity and cell death [40, 264–266]. GD is known to induce the production of mitochondrial ROS, O_2_^−^, and intracellular H_2_O_2_ [71, 260]. GD actually induces necrosis through the production of ROS [40]. Inhibition of ROS production by N-acetyl-L-cysteine (NAC) and catalase prevents GD-induced necrosis and switches the cell death mode to apoptosis [40].

Metabolic stress induces the loss of mitochondrial membrane potential and mitochondrial permeability transition (mPT). ROS can induce mPT in the mitochondrial inner membrane, while the mPT pore opening can induce apoptosis by causing the release of mitochondrial apoptotic molecules. Prolonged opening of the membrane results in necrotic cell death [262]. The mitochondrial membrane potential (ΔΨm) is also lost upon the mPT pore opening. If the mPT pore is open for longer periods, cells cannot generate ATP by oxidative phosphorylation, leading to necrotic cell death as a consequence of ATP depletion. These data suggest that GD-induced necrosis is implicated in excess ROS production, mitochondrial dysfunction, and ATP depletion.

OGD has been shown to increase ROS production, which can induce necrosis in solid tumors [8, 54]. Furthermore, OGD has been shown to disrupt mitochondrial function, causing oxidative stress and necrosis in various cancer cells and cultured myocardial cells. Hypoxia-ischemia induces anaerobic metabolism and lactate accumulation, resulting in decreased ATP levels and intracellular pH. Consequently, intracellular and mitochondrial calcium levels and cell swelling and rupture are increased due to dysfunction of ATPase-dependent ion transport mechanisms. Thus, cell death processes such as necrosis and apoptosis are triggered [267].

In contrast to GD-induced necrosis, GD-induced apoptosis is ROS-independent. GD-induced ceramide enhances TRAIL-induced apoptosis through the downregulation of FLIP by the dephosphorylation of Akt in human prostate adenocarcinoma DU-145 cells. Consequently, GD enhances TRAIL-induced apoptotic cell death [268]. In addition, 2-deoxyglucose (2-DG), a nonmetabolizable glucose analog that is frequently used to mimic glucose starvation, induces apoptosis including apoptotic nuclear morphology and caspase activity, whereas GD induces necrosis [269]. Moreover, the transcription factor ATF4 is involved in the apoptosis induced by 2-DG as well as the necrosis provoked by GD. Several hexoses partially prevent GD-induced necrosis in rhabdomyosarcoma, although only mannose prevents 2-DG-induced apoptosis. The reduction of GD-induced necrosis as well as 2-DG-induced apoptosis was related with decreased ATF4 levels [269].

In low-density culture, cortical neurons die by necrosis, whereas they die by apoptosis in high-density culture. Conditioned medium factors prepared from the high-density culture switch the cell death mechanism from necrosis to apoptosis through a PKC-dependent signaling pathway [270]. Hypoglycemia induces necrotic cell death by transcription factor CEBP homology protein (CHOP) in neuroblastoma cells, and the cells evade death by repressing CHOP and inducing VEGF upon hypoxia [271]. The induction of necrosis in response to GD is blocked by MUC1 oncoprotein, which is aberrantly overexpressed in most human carcinomas [272]. MUC1 may suppress GD-induced ROS increases, thereby inhibiting ATP depletion and cell death processes such as autophagy. In addition, MUC1 enhances lysosomal turnover of LC3-II, a marker of autophagy, and then promotes autophagy through an AMPK-dependent mechanism [272]. MUC1 expression is known to be regulated by the EGFR (epidermal growth factor receptor) signaling pathway, which can be activated by extracellular matrix protein 1 (ECM1), a secreted glycoprotein overexpressed in various tumors. Additionally, MUC1 has been shown to be involved in ECM1-induced EMT, metastasis, and the Warburg effect through enhancing *β*-catenin expression at the posttranslational level, which alters the gene expression that potentiates EMT progression and the CSC phenotype [273–275].

In HUVECs, OGD leads to mitochondrial necrosis via mitochondrial p53-cyclophilin D (Cyp-D) association, mitochondrial depolarization, ROS production, and lactate dehydrogenase (LDH) breach. AMPK activation is known to be inhibited by microRNA-451. AntagomiR-451, a microRNA-451 inhibitor, inhibits OGD-induced programmed necrosis by AMPK activation in HUVECs [276].

#### 5.2.2. Role of Dlx-2/Snail Cascade in GD-Induced Necrosis in Tumors

Phorbol-12-myristate-13-acetate (PMA), a PKC activator, prevents GD-induced necrosis and switches the cell death mode to apoptosis [40]. To identify OGD-induced necrosis-linked molecules, we conducted gene expression profiling by analyzing cDNA microarrays in the case of A549 cells with or without cotreatment with GD and phorbol-12-myristate-13-acetate (PMA), a protein kinase C (PKC) activator, that can switch the cell death mode from necrosis to apoptosis. Without treatment, necrosis occurred, while apoptosis but not necrosis was found upon cotreatment [71, 260]. Of 3096 genes analyzed, approximately 200 were upregulated >2-fold and approximately 150 were downregulated >2-fold (GEO accession number GSE24271), indicating that gene expression patterns changed during necrotic cell death. Target genes for the regulation of necrosis were identified, such as ANKRD1, GEM, FOS, SGK, IL-8, RGS2, IL-2, CD5L, CTGF, MAS1, and others. Among them, Snail, distal-less homeobox-2 (Dlx-2), and early growth response-1 (Egr-1) were induced in cells that undergo necrosis but not in those that died by apoptosis (GEO accession number GSE24271) [71, 260, 277].

Snail, Dlx-2, and Egr-1 are potential OGD-induced necrosis-linked genes. In fact, Snail, Dlx-2, and Egr-1 were induced in cells that undergo necrosis [71, 260, 277]. The molecular mechanisms of OGD-induced necrosis focus on Snail, Dlx-2, and Egr-1. Snail is a zinc finger transcription factor that induces epithelial-mesenchymal transition (EMT) by directly repressing E-cadherin expression. Snail expression can be induced by many kinds of tumor-stimulating cytokines, such as transforming growth factor- (TGF-) *β*, Wnt, Notch, and hedgehog, in many human invasive carcinomas [171, 278–285]. In addition, Snail protects cells from apoptosis induced by the withdrawal of survival factors or proapoptotic stimuli [286–290].

Dlx-2 is also a transcription factor originally known to be involved in embryonic development, tissue homeostasis, and the cell cycle [291, 292]. Recently, it is regarded to play an important role in carcinogenesis, since Dlx-2 expression correlates with more advanced cancer stage and with poor prognosis in a variety of human cancer types [260, 293–295].

Egr-1 is induced by hypoxia and plays a critical role in hypoxia-induced tumor progression, survival, and angiogenesis [296–299]. Furthermore, Egr-1 is involved in hepatocyte growth factor- (HGF-) induced cell scattering, migration, and invasion via Snail activation [300]. While transient induction of Egr-1 is known to activate angiogenesis, sustained Egr-1 expression induces antiangiogenesis, growth arrest, and apoptosis [301]. Thus, Egr-1 is thought to act as a crucial regulator of tumor cell death, growth, invasion, and angiogenesis.

We previously showed that Snail expression is increased by Dlx-2, which acts as an upstream regulator of Snail. TGF-*β* and Wnt induce the expression of Snail in a Dlx-2-dependent manner [302, 303]. In addition, IR has been shown to upregulate Dlx-2 by activating Smad2/3 signaling in A549 and MDA-MB-231 cell lines [304]. The induction of Snail, Dlx-2, and Egr-1 in response to GD is regulated in a ROS-dependent manner [71, 260], and H_2_O_2_ (300 *μ*M) or menadione (10 *μ*M, an O_2_^−^ generator) treatment increases the expression of these proteins [71, 260, 277]. Consistent with this, PI3K/Akt-mediated inhibition of GSK3*β* is responsible for Snail expression mediated by ROS such as H_2_O_2_ in MCF-7 cells [305]. Similarly, ROS-dependent Snail induction has been reported in hepatocellular carcinoma cells [305] and MMP-3/Rac1b signaling-mediated EMT of mouse mammary epithelial cells [306]. Metabolic stress-induced Snail, Dlx-2, and Egr-1 expression was also detected in the innermost region of MTS and its middle regions that are likely to exhibit hypoxic conditions [71, 260, 277].

In fact, hypoxia is known to induce Snail mRNA expression in ovarian cancer cell lines [307]. Snail expression under hypoxia has been suggested to be mediated by hypoxia-inducible factor 1 (HIF1) itself as well as HIF1-TGF-Smad signaling [308]. Moreover, there is another mechanism controlling Snail protein levels. Hypoxia increases the levels of Snail protein through HIF-1/Twist-dependent downregulation of F-box E3 ubiquitin ligase (FBXl14) expression, which promotes Snail ubiquitinylation and proteasome degradation independently of phosphorylation by GSK-3*β* [309]. Hypoxia also induces Snail translocation into the nucleus through ROS-dependent inhibition of GSK-3*β* [310, 311].

Knockdown of Snail, Dlx-2, or Egr-1 using specific shRNA inhibits metabolic stress-induced necrosis and the release of HMGB1 and lactate dehydrogenase (LDH) [71, 260]. In addition, knockdown of Snail switches GD-induced necrosis to autophagy-like cell death but not apoptosis. In addition, knockdown of Dlx-2 switches GD-induced necrotic cell death to apoptosis [260]. The antinecrotic effects of Snail shRNA, Dlx-2 shRNA, or Egr-1 shRNA were also observed in MTS, indicating that the expression of Snail, Dlx-2, and Egr-1 is related to microenvironmental stresses such as hypoxia and GD [71, 260, 277]. These results indicate that Snail/Dlx-2/Egr-1 is a crucial regulator of GD-induced necrosis [71, 260, 277].

Snail knockdown also inhibits MTS growth. Snail has been shown to block the cell cycle [287], and Snail-overexpressing cells exhibit significantly longer population doubling times than vector control cells (33.8 h and 27.7 h, resp., data not shown). Thus, Snail shRNA-mediated MTS growth inhibition cannot be explained by the effects of Snail on the cell cycle and may occur via a unknown mechanism of Snail shRNA other than its effects on the cell cycle. Even though Snail shRNA MTS exhibited smaller sizes at day 9 (453 ± 30 mm), the size is likely to be enough to result in metabolic stress in core regions, because 9-day Snail shRNA MTS underwent apoptotic cell death (instead of necrosis). In fact, necrotic cores form in most spheroids larger than 400–500 mm [71, 312]. In the case of MCF-7 MTSs, while preventing necrosis, Snail shRNA switched the cell death mode to apoptosis [71].

In addition, Dlx-2 or Egr-1 silencing in MCF-7 MTSs slightly suppresses the growth of MCF-7 MTSs and prevents metabolic stress-induced necrosis in MTS [260, 277]. Thus, Snail, Dlx-2, and Egr-1 seem to be implicated in GD-induced necrosis and tumor progression.

#### 5.2.3. How Do Snail and Dlx-2 Control Mitochondrial Activity?

Mitochondrial ROS are closely linked to GD-induced cytotoxicity and cell death [40, 264–266]. GD is known to contribute to the production of mitochondrial ROS, O_2_^−^, and intracellular H_2_O_2_ and also triggers necrotic cell death [71, 260, 277].

Mitochondrial dysfunction has been linked to the induction of necrosis. Tumor cells have been shown to exhibit abnormal mitochondrial structure and DNA integrity and high rates of mtDNA mutations [313, 314], and this has been suggested to sensitize the cells to oxidative stress and cell death induced by GD or treatment with 2-DG [266]. In addition, tumor cells with dysregulated mitochondria undergo necrosis instead of apoptosis in response to alkylating DNA damage, which induces rapid ATP depletion through PARP activation [36].

Metabolic stress-induced Snail protein aggregates are colocalized with the mitochondria, possibly in their inactive form in which transcriptional activity is impaired [315]. Thus, Snail aggregates may affect mitochondrial function and sensitize tumor cells to metabolic stress and death by necrosis. Knockdown of Snail, Dlx-2, or Egr-1 blocks GD-induced production of intracellular ROS, indicating that Snail, Dlx-2, and Egr-1 may control necrosis by regulating metabolic stress-induced mitochondrial ROS production [71, 260, 277].

ROS produced under stress conditions are known to spread from one mitochondrion to neighboring mitochondria in a process known as ROS-induced ROS release (RIRR), constituting a positive feedback mechanism for enhanced ROS production, leading to mitochondrial and cellular injury [316, 317]. The induction of mitochondrial ROS up to a critical threshold level has been suggested to be a key step in propagating the synchronized RIRR response.

ROS can induce insoluble protein aggregates that are toxic to cells and cause cell death, especially necrosis, through triggering necrosis-associated membrane rupture. Interestingly, GD-induced Snail protein remains in the cytosol and forms insoluble aggregates with proapoptotic molecules p53, caspase-3, and caspase-9 and the proautophagic molecule Beclin-1, in a ROS-dependent manner during GD-induced necrosis [315]. A similar pattern of protein aggregates has been demonstrated in focal ischemic regions of brain tissue, which is similar to the OGD region found in tumors [318–320]. Ring-like structures among protein aggregates (oligomeric globular assemblies, protofibrils, and ring-like structures) can form nonspecific membrane pores that lead to necrosis [321].

In addition, Snail knockdown may exert its antinecrotic effects through preventing metabolic stress-induced loss of mitochondrial membrane potential, mitochondrial permeability transition, and metabolic stress-induced protein aggregation, which are the primary events that trigger necrosis, by inhibiting mitochondrial ROS production [71, 315]. Similarly, knockdown of Dlx-2 or Egr-1 also prevents metabolic stress-induced mitochondrial ROS production, loss of mitochondrial membrane potential, and mitochondrial permeability transition [260, 277].

**Figure 4:**
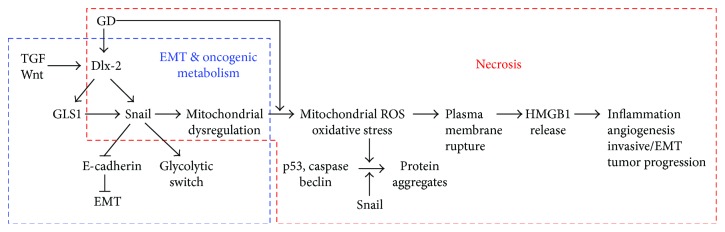
Snail and Dlx-2 regulate metabolic stress-induced necrosis in tumors by inducing EMT, mitochondrial dysregulation, and oncogenic metabolism. GD-induced Snail and Dlx-2 may cause mitochondrial dysfunction, facilitating ROS production in response to GD. Increased ROS can induce insoluble protein aggregates containing p53, caspase, and beclin and cause necrosis through triggering the plasma membrane rupture and HMGB1 release. In addition, metabolic stress-induced necrosis is driven by increased ROS, which is stimulated by Snail and Dlx-2, which mediates EMT for tumor invasion in the absence of metabolic stress. The Dlx-2/GLS1/glutamine metabolic axis can regulate TGF-*β*/Wnt-induced, Snail-dependent EMT, and glycolytic switch. Metabolic stress-induced Snail and Dlx-2 expression contributes to tumor progression by promoting necrosis as well as by inducing EMT and oncogenic metabolism.

Therefore, GD-induced Snail (including the aggregated form), Dlx-2, or Egr-1 expression may cause mitochondrial dysfunction, facilitating ROS production in response to GD. This increased ROS level may in turn enhance Snail, Dlx-2, or Egr-1 expression to accelerate massive ROS production by RIRR and to induce GD-induced cytotoxicity and necrosis, thereby forming a positive feedback loop between Snail, Dlx-2, or Egr-1 expression and cellular ROS levels [71, 260]. These results indicate that Snail, Dlx-2, Egr-1 are implicated in metabolic stress-induced necrosis in tumor progression. The mechanisms of Snail-, Dlx-2-, or Egr-1-mediated necrosis appeared to be related to mitochondrial ROS production, loss of mitochondrial membrane potential, and mitochondrial permeability transition, which are the primary events that trigger necrosis (Figure 4).

## 6. Regulation of Oncogenic Metabolism by Necrotic Regulators Dlx-2 and Snail

Snail, Dlx-2, and Egr-1 have the potential to regulate mitochondrial activity. In fact, Snail, Dlx-2, and Egr-1 have been shown to induce mitochondrial repression and glucose metabolism by downregulating cytochrome C oxidase (COX) subunits or fructose-1,6-bisphosphatase 1 (FBP1) [198, 199, 277, 302, 303, 322]. Thus, oncogenic metabolism in cancer cells is closely associated with necrosis.

The cells in the inner regions of tumors experience exacerbated OGD and induce necrotic cell death through metabolic reprogramming.

### 6.1. Oncogenic Metabolism

Several signaling pathways (such as those of TGF-*β*, Wnt, EGF, Hedgehog, Notch, and ROS) can activate oncogenes and inactivate tumor suppressors, thereby inducing tumorigenesis and tumor progression. Cancer cells can acquire multiple biological capabilities during their multistage development. Hanahan and Weinberg proposed ten hallmarks of cancer that alter cell physiology to enhance malignant growth: (1) sustained proliferation, (2) evasion of growth suppression, (3) cell death resistance, (4) replicative immortality, (5) evasion of immune destruction, (6) tumor-promoting inflammation, (7) activation of invasion and metastasis, (8) induction of angiogenesis, (9) genome instability, and (10) alteration of metabolism [210, 323]. Among these ten hallmarks, metabolic reprogramming may be required for malignant transformation and tumor development, including invasion, metastasis, necrosis, and EMT [324].

Most cancer cells produce their energy predominantly by highly utilizing glycolysis rather than oxidative phosphorylation, even in the presence of oxygen, a phenomenon that has been termed the Warburg effect, aerobic glycolysis, or the glycolytic switch [325–334]. These alterations contribute to cell survival and sustain the increased demands of cell proliferation by providing biosynthetic precursors for nucleic acids, lipids, and proteins. Other oncogenic metabolic pathways, including glutamine metabolism, the pentose phosphate pathway (PPP), and synthesis of fatty acids and cholesterol, are also enhanced in many cancers [326–336]. Several transcription factors, including HIF-1*α*, p53, and c-Myc, are known to contribute to oncogenic metabolism [326–334].

### 6.2. Oncogenic Metabolism and Necrosis

The activation of oncogenes and the loss of tumor suppressors have been shown to drive tumor progression; in particular, they seem to drive metabolic reprogramming. This suggests that metabolic reprogramming in cancer cells is closely associated with tumor growth and proliferation [326–334]. Emerging evidence suggests that metabolic reprogramming is a hallmark of cancer and may be required to convert a normal cell into a malignant cell [326–334]. Tumor cells with dysregulated mitochondria and the glycolytic switch have been shown to exhibit apoptotic resistance [337] and necrosis that may promote tumor progression and aggressiveness upon metabolic stress [8, 9].

Cancer cells may overcome oxygen and nutrient deprivation by reprogramming their metabolism, for example, by highly increasing anaerobic glycolysis, which contributes to tumor growth and drug resistance. GLUTs are increased abnormally and colocalize with HIF1*α* in perinecrotic regions in human colorectal carcinoma [338]. Under hypoxic conditions, RIP1/3 complex formation and phosphorylation increase in glucose-free media, but this does not occur under normoxic conditions. RIP-dependent necroptosis in hypoxic cancer is suppressed by glycolytic pathways, including pyruvate, which scavenges mitochondrial superoxide without restoring cellular energy [338].

Metabolic alterations may contribute to malignant transformation and tumor development, including the induction of EMT, invasion, metastasis, and TME [303, 322, 339–344]. Dlx-2 and Snail are implicated in oncogenic metabolism. We previously showed that the Dlx-2/Snail cascade suppressed mitochondrial respiration and cytochrome c oxidase (COX), the terminal enzyme of the mitochondrial respiratory chain. Dlx-2/Snail cascade also induces glycolytic switch [198, 302].

In addition, Dlx-2 and Snail expression are induced by TGF-*β* and Wnt and regulate TGF-*β*- and Wnt-induced glycolytic switch. TGF-*β*/Wnt suppresses mitochondrial respiration and COX activity in a Dlx-2/Snail-dependent manner. TGF-*β*/Wnt appeared to downregulate the expression of various COX subunits, including COXVIc, COXVIIa, and COXVIIc [302]. Thus, the Dlx-2/Snail axis is important in TGF-*β*/Wnt-dependent glycolytic switch.

Using cDNA microarray technology, we also found that Dlx-2 upregulated several metabolic enzymes, including GLS1, PFKFB2, H6PD, and ACACB, which are involved in Gln metabolism, glycolysis, the PPP, and fatty acid/cholesterol synthesis, respectively, suggesting that Dlx-2 may activate several oncogenic metabolic pathways (the microarray dataset is available in GSE61009).

Glutamine metabolism plays an important role in inducing EMT [303]. Glutaminase 1 (GLS1) converts glutamine to glutamate, which acts as an intermediate in the TCA cycle. GLS1 is known to be an important regulator of Snail-induced EMT and is also a metabolism-linked target gene of Dlx-2, contributing to tumor progression. Inhibiting glutamine metabolism (via GLS1 knockdown, glutamine deprivation, or glutamine metabolism inhibitors) suppresses Dlx-2-, TGF-*β*-, Wnt-, and Snail-induced EMT. In addition, GLS1 knockdown also suppresses tumor growth and metastasis *in vivo*. Dlx-2 knockdown and glutamine metabolism inhibition decrease Snail mRNA levels through the p53-dependent upregulation of Snail-targeting microRNAs. These results indicate that the Dlx-2/GLS1/glutamine metabolic axis is a crucial regulator of TGF-*β*/Wnt-induced, Snail-dependent EMT and metastasis [303]. Oncogenic metabolism, including glutamine metabolism, is known to endow cancer cells with growth advantages by providing biosynthetic precursors [327–336]. Given that GLS1 knockdown suppresses tumor growth and metastasis *in vivo*, it is possible that knocking down any component enzyme in oncogenic metabolism results in a pronounced suppression of metastasis. Like GLS1, other oncogenic metabolism enzymes may also regulate p53-dependent modulation of Snail-targeting microRNAs to mediate Snail-induced EMT.

In addition, inhibiting glutamine metabolism (via GLS1 knockdown, glutamine deprivation, or glutamine metabolism inhibitors) suppresses the Dlx-2-, TGF-*β*-, Wnt-, and Snail-induced glycolytic switch. These results indicate that the Dlx-2/GLS1/glutamine metabolic axis is a crucial regulator of the TGF-*β*/Wnt-induced, Snail-dependent glycolytic switch [303]. These results suggest that other oncogenic metabolism enzymes, including GLS1, may also regulate the TGF-*β*/Wnt-induced, Dlx-2/Snail-dependent glycolytic switch.

Oncogenic metabolism is not only associated with the EMT, invasion, and metastasis but also contributes to necrosis. Many regulatory molecules involved in necrosis, including Snail and Dlx-2, are implicated in the metabolic reprogramming of cancer cells [198, 199, 302, 303, 322, 345–350]. Metabolic stress-induced necrosis is driven by increased ROS production that is stimulated by Snail and Dlx-2, which mediates EMT for tumor invasion in the absence of metabolic stress. Accordingly, Snail and Dlx-2 contribute to tumor progression by promoting necrosis as well as by inducing EMT and oncogenic metabolism (Figure 4).

c-Myc is implicated in necrosis. The expression of c-Myc protein, which participates in energy-consuming processes such as proliferation and ribosome biosynthesis, is often dysregulated in human cancers [351]. The reduced availability of oxygen and glucose results in the rapid reduction of c-Myc protein levels, mainly due to enhanced degradation. OGD-induced necrosis is restrained by c-Myc knockdown via shRNA. Presumably, an environmental milieu that controls c-Myc protein levels, particularly those that downregulate c-Myc, might be a strategy for cancer cells to survive with limited energy sources [351].

## 7. Conclusion

The cells in the inner regions of the solid tumors display hypoxia and/or higher rates of aerobic glycolysis, which occurs because of insufficient blood supply. Thus, these changes may exacerbate oxygen and glucose deprivation and induce necrotic death [1, 3, 4, 64]. Necrosis was previously considered an accidental and genetically unprogrammed form of cell death. Necrosis begins with cell swelling, resulting in cell membrane rupture and the release of cellular cytoplasmic contents into the extracellular space, such as the proinflammatory and tumor-promoting cytokine, HMGB1 [5, 9–13]. As the best-known and characterized DAMP molecule, HMGB1 stimulates inflammation and angiogenesis to promote tumor progression [77–80]. Recently, necrosis has been recognized as a programmed cell death, such as necroptosis, and others. In addition, the mechanism of OGD-induced necrosis was recently verified by the role of Dlx-2/Snail. Many regulatory molecules involved in necrosis, including Snail and Dlx-2, are implicated in the metabolic reprogramming of cancer cells [198, 199, 302, 303, 322, 345–350]. Snail and Dlx-2 have been shown to induce mitochondrial repression and glucose metabolism by downregulating cytochrome C oxidase (COX) subunits or fructose-1,6-bisphosphatase 1 (FBP1) [198, 199, 302, 303, 322]. Thus, oncogenic metabolism in cancer cells is closely associated with necrosis. The cells in the inner regions of tumors exhibit exacerbated oxygen and glucose deprivation (OGD) and induce necrotic death through metabolic reprogramming. OGD-induced necrosis plays an important role in tumor progression; however, its regulatory mechanisms have been poorly investigated. Cancer cells ideally induce cell cycle inhibition or apoptotic death in response to cytotoxic agents. However, excessively high intensity of specific agents induces necrotic cell death [352, 353]. In general, the mode of cell death can change depending on the drug concentration; necrosis occurs with high doses and apoptosis with lower doses [353]. Drug-resistant cells such as MCF-7/R (DOX-resistant) respond to chemotherapy by undergoing necrosis or late apoptosis, whereas drug-nonresistant MCF-7/W (wild-type) cells respond by undergoing early and then late apoptosis. Necrosis is correlated with poor prognosis because it triggers inflammation and promotes tumor growth. Thus, drug resistance has been considered a fundamental problem in the chemotherapeutic treatment of most common human cancers [352]. Actually, both drug-resistant and nonresistant cells respond to DOX conjugates based on 4-arm stars by showing apoptosis without necrosis [352].

Taken together, necrosis might be largely related to side effects of anticancer therapies. Anticancer agents targeting apoptosis frequently induce excessive or unwanted effects even at therapeutic doses. However, most studies of anticancer drugs have focused on apoptosis, although necrosis has important clinical implications in chemotherapy. Necrosis should be emphasized as well as apoptosis to improve cancer treatment [354]. Thus, understanding the molecular mechanisms of metabolic stress-induced necrosis in tumors is necessary to our knowledge of its role in tumor development and will be crucial for the development of therapeutic strategies.
